# Building the Framework for Standardized Clinical Laboratory Reporting of Next-generation Sequencing Data for Resistance-associated Mutations in *Mycobacterium tuberculosis* Complex

**DOI:** 10.1093/cid/ciz219

**Published:** 2019-03-18

**Authors:** Jeffrey A Tornheim, Angela M Starks, Timothy C Rodwell, Jennifer L Gardy, Timothy M Walker, Daniela M Cirillo, Lakshmi Jayashankar, Paolo Miotto, Matteo Zignol, Marco Schito

**Affiliations:** 1 Division of Infectious Diseases, Johns Hopkins University School of Medicine, Baltimore, Maryland; 2 Division of Tuberculosis Elimination, National Center for HIV/AIDS, Viral Hepatitis, STD, and TB Prevention, Centers for Disease Control and Prevention, Atlanta, Georgia; 3 Foundation for Innovative New Diagnostics, Geneva, Switzerland; 4 Division of Pulmonary, Critical Care, and Sleep Medicine, University of San Diego, California; 5 School of Population and Public Health, University of British Columbia, Canada; 6 Clinical Prevention Services, British Columbia Centre for Disease Control, Vancouver, Canada; 7 Nuffield Department of Medicine, University of Oxford, United Kingdom; 8 IRCCS San Raffaele Scientific Institute, Milano, Italy; 9 Columbus Technologies, Inc. Contractor to the National Institute of Allergy and Infectious Diseases, US National Institutes of Health, Bethesda, Maryland; 10 Global TB Programme, World Health Organization, Geneva, Switzerland; 11 Critical Path to Tuberculosis Drug Regimens, Critical Path Institute, Tucson, Arizona

**Keywords:** tuberculosis, next-generation sequencing, reporting, standardization, interpretation

## Abstract

Tuberculosis is the primary infectious disease killer worldwide, with a growing threat from multidrug-resistant cases. Unfortunately, classic growth-based phenotypic drug susceptibility testing (DST) remains difficult, costly, and time consuming, while current rapid molecular testing options are limited by the diversity of antimicrobial-resistant genotypes that can be detected at once. Next-generation sequencing (NGS) offers the opportunity for rapid, comprehensive DST without the time or cost burden of phenotypic tests and can provide useful information for global surveillance. As access to NGS expands, it will be important to ensure that results are communicated clearly, consistent, comparable between laboratories, and associated with clear guidance on clinical interpretation of results. In this viewpoint article, we summarize 2 expert workshops regarding a standardized report format, focusing on relevant variables, terminology, and required minimal elements for clinical and laboratory reports with a proposed standardized template for clinical reporting NGS results for *Mycobacterium tuberculosis*.

## Teaser

An expert consensus process was used to develop a standardized framework for reporting and communicating clinically relevant next-generation sequencing data from *Mycobacterium tuberculosis* for guiding drug treatment options.

Tuberculosis is a critical global health concern, with an estimated 10 million new cases in 2017, including approximately 460 000 multidrug-resistant (MDR) cases (resistant to isoniazid and rifampin) [1]. MDR-tuberculosis rates are estimated to rise in 4 high-burden countries due to ongoing transmission, raising the stakes on identification and resistance profiling of new cases [2]. While culture-based phenotyping remains the reference standard for drug susceptibility testing (DST), the paradigm has shifted recently with the World Health Organization (WHO) endorsement and global implementation of molecular assays such as Cepheid Xpert® MTB/RIF [3] and line-probe assays [4, 5] that provide rapid DST results without the time or cost burden of phenotypic testing. While transformative, existing rapid molecular tests are limited by the diversity of resistant genotypes that can be detected simultaneously. Whole-genome sequencing (WGS) and targeted DNA sequencing directly from clinical samples offer potential comprehensive solutions for rapid diagnosis, DST, and large-scale drug-resistance surveillance [6–8]. In 2017, Public Health England implemented WGS for all mycobacterial cultures, and the United States announced plans for universal WGS surveillance, while New York State’s Wadsworth Center has incorporated WGS since 2016 [9–11]. The European Centre for Disease Prevention and Control is also standardizing WGS across the 28 member states to monitor cross-border transmission, and WHO has led multicountry population-level surveys to determine the best use of next-generation sequencing (NGS) and DST worldwide, laying the groundwork for widespread access [8, 12, 13]. Current sequencing programs often rely on centralized government or academic facilities due to the need for specially skilled laboratorians, infrastructure, data management capacity, and bioinformatics. Efforts to overcome these obstacles and aid broader NGS deployment are underway and will likely provide global access to DNA sequencing technologies for clinical diagnosis of drug-resistant tuberculosis in the next 5 years [14–17].

Increasing global reliance on molecular assays for tuberculosis requires improved laboratory reporting language to ensure that results are communicated clearly, consistently, and comparably across laboratories and are associated with interpretative guidance for clinical decision making. Such a framework would help standardize implementation of effective treatment guidelines and improve confidence in results [18]. Although several institutions have implemented individualized NGS reporting schemes, a globally developed framework for clinical reporting of molecular DST results for *Mycobacterium tuberculosis* (*Mtb*) is needed to standardize interpretation and clinical application worldwide. The TBNET and RESIST-TB networks have provided perspectives on reporting standards, including a review of common mutations, associations with resistance, endorsement of specific mutations to report, and interpretative guidance to help select effective treatment [19, 20]. Additional efforts such as the Relational Sequencing TB Data Platform (ReSeqTB) [21] are standardizing analytic and interpretative criteria for drug-resistance determining mutations, and WHO recently released a technical guide on NGS for *Mtb* [22]. However, a harmonized reporting format is essential to ensure that patients across the globe reap similar benefits from the same sequencing results.

To address this need, the Critical Path Institute (C-Path) in collaboration with WHO and the US National Institutes of Health, Division of AIDS, National Institute of Allergy and Infectious Diseases, convened a workshop in London, United Kingdom, 3–4 February 2016, followed by a second workshop cosponsored by C-Path and FIND diagnostics on 27–28 September 2017. The objectives were to produce a proposed standardized reporting format and to define comprehensive and minimal data elements to include in clinical and laboratory reports of NGS data. In this Viewpoint article, we describe key discussion points and consensus outcomes regarding clinical reporting of NGS-derived molecular DST results for *Mtb*.

## CHALLENGES AND OPPORTUNITIES FOR MOLECULAR DST

Diagnostic devices that incorporate genomics are increasingly being deployed globally. A recent publication showed that WGS can predict antimicrobial resistance to first-line drugs with high sensitivity that, if confirmed, will likely reduce phenotypic testing in low-burden countries [23]. In addition, sequence data provide richer information than the categorical “susceptible” vs “resistant” clinical results most often reported from molecular diagnostics. Thus, sequence data have the potential to be used for quantitative interpretations (eg, as needed for transmission tracking or identifying heteroresistance) and more nuanced clinical conclusions (eg, specific mutations associated with specific elevations in minimum inhibitory concentration [MIC]), which could ultimately enable more sophisticated treatment decision support. NGS pipelines, including the ReSeqTB data platform and PhyResSE, are standardizing the bioinformatics approaches and catalogues of confidence-graded resistance-associated mutations required for molecular DST. However, even the best assays can still produce discordant results between molecular and growth-based DST [21, 24, 25]. Challenges include an evolving consensus on specific loci of interest, a constantly improving knowledge base for associating detected mutations with drug resistance, variable numbering systems for relevant genes, assay limits of detection, interpretation of heteroresistance, limited bioinformatics capacity in the regions of highest need, and lack of a global reporting standard.

Although most drug-resistance mechanisms are well documented for *Mtb*, several knowledge gaps remain. WGS can be used to evaluate substantially more loci than polymerase chain reaction assay or targeted sequencing, and accurate susceptibility prediction for each mutation requires a large dataset of isolates with comprehensive phenotypic DST. As a result, it is difficult to reliably associate drug resistance with uncommon mutations, insertions and deletions (indels), and mutations that affect drugs for which susceptibility is rarely tested. In addition, there is no standard nomenclature for mutation reporting in infectious disease microbiology, complicating reports for genes such as *rpoB* that have distinct numbering systems for *Escherichia coli* and *Mtb*. Use of a consensus numbering system would ensure consistent interpretation of these mutations [26]. Uniform standards such as those used by molecular pathology and medical genetics organizations could help improve clarity of *Mtb* reporting [27–30].

Even when these problems are resolved, NGS-based DST will still require significant bioinformatic programming written and maintained by highly trained staff. Automated, cloud-based platforms such as PhyResSE and ReSeqTB have integrated unified variant pipelines for use by laboratories with less bioinformatics expertise in order to generate variant reports with clinical interpretation [21, 25]. Similar WGS pipelines could be developed to analyze lineage and strain relatedness for molecular epidemiology studies, transmission mapping, and identification of potential outbreaks, especially in low-incidence settings such as the pipeline used by Public Health England [31]. While these online services are still being developed for full public access, they are positioned to transform the analysis of NGS data and democratize the utility of this technology for nonexpert use.

As NGS becomes widespread in programmatic environments worldwide [23], a global standard for recording, reporting, and interpreting these data must grow in parallel, so that globally representative data can be collated for both DST and surveillance purposes [8]. Effective clinical implementation of NGS should classify mutations using a robust statistical approach in order to understand the predictive value for drug resistance as determined by correlation with growth-based DST results and, ideally, clinical outcomes. This should align with standardized reporting efforts to generate reports that healthcare providers and public health programs can easily understand. For *Mtb*, a standardized classification scheme for clinically grading mutations in *Mtb* was proposed using likelihood ratios, similar to clinical decision-making tools used in evidence-based medicine [20]. However, the value of mutation grading schemes will greatly depend on the quality of the dataset used for these calculations and the frequency of mutations in that dataset. The addition of MIC ranges to resistance prediction could also aid healthcare providers in clinical management. Highly curated, dynamic databases such as ReSeqTB [21] and projects such as the Comprehensive Resistance Prediction for Tuberculosis: an International Consortium [23, 32] generate high-quality phenotypic and WGS data for large numbers of well-characterized isolates. Ideally, a laboratory report could leverage these large datasets to interpret and classify mutations identified in clinical samples in order to allow healthcare providers to devise effective treatment regimens. Initiatives to develop interoperable database ontologies for consolidating drug resistance in *Mtb* will allow reports to be updated as datasets are standardized and integrated to interpret additional mutations and improve confidence in resistance predictions over time.

## DEVELOPMENT OF STANDARDIZED REPORTS

A standardized format and nomenclature for reporting NGS results must balance simplicity and clarity while providing the necessary data required by healthcare providers, country-level surveillance programs, and stringent regulatory authorities that seek to regulate NGS as an in vitro diagnostic. The report should be flexible to the needs of users with different training levels, with a simple summary, additional details for expert users, and a flexible structure to grow as resistance-predicting datasets expand. To meet these needs, the authors designed example laboratory reports in consultation with the February 2016 workshop participants and circulated them to dozens of stakeholders worldwide. After multiple revisions, report templates were presented at the September 2017 workshop.

Meeting participants included researchers, laboratorians, NGO representatives, clinicians, bioinformaticians, epidemiologists, government representatives, and public health workers, with representatives from Europe, North and South America, Southeast Asia, Africa, and the Western Pacific. A live polling tool incorporated participants’ feedback on each data element, which was combined with design study research principles to generate these reports [33]. A strong preference emerged for a laboratory report that healthcare providers and laboratorians with a range of education levels and expertise could use for clinically relevant drug-resistance prediction. Meeting participants discussed that molecular epidemiology data would be desirable in some circumstances, but including transmission cluster analysis was not identified as a priority for the report template given the focus on drug resistance. The intended purpose of these reports is to provide information that is useful for healthcare providers in determining optimal treatment regimens. Workshop participants concluded that results should be presented as 2 reports: a 1-page simplified report for healthcare providers and laboratorians with less expertise in NGS and a longer-form report for providing data on a comprehensive set of NGS variables.

### Proposed Reporting Formats

**Figure 1.**
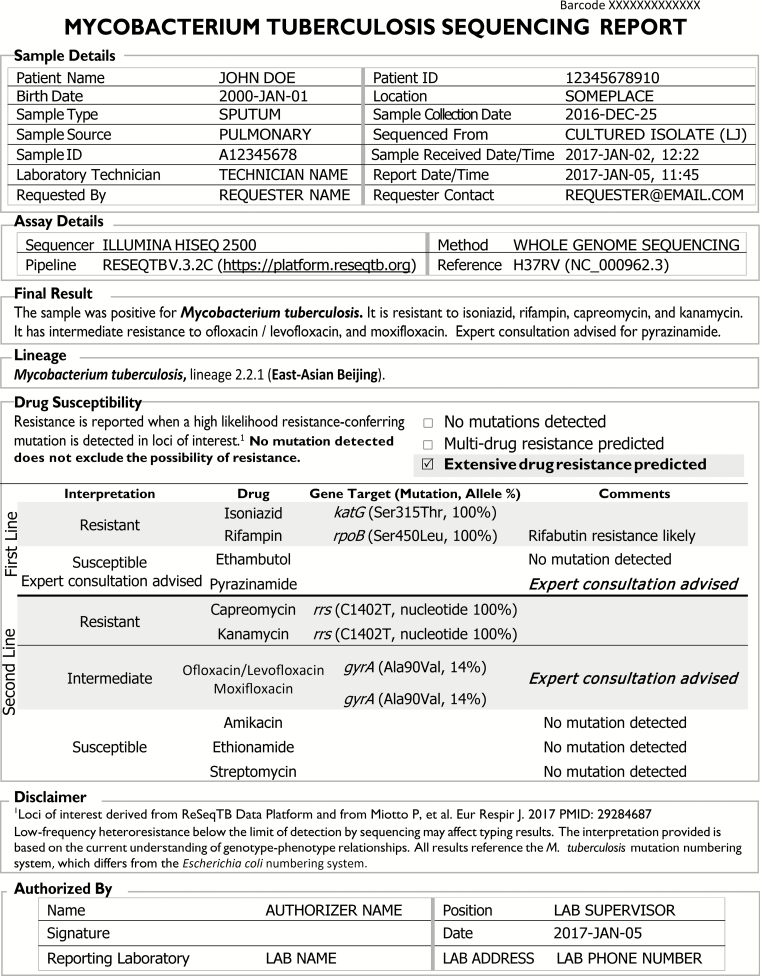
One-page *M. tuberculosis* sequencing summary report to assist in making clinical decisions for patient management.

**Figure 2.**
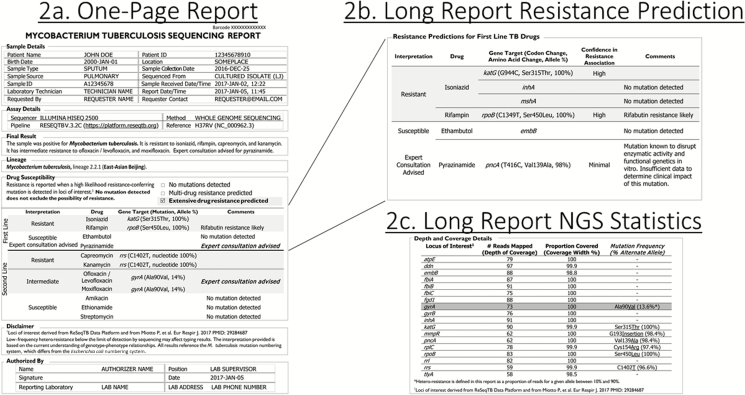
Detailed multi-page *M. tuberculosis* laboratory sequencing report containing additional information to aid in interpretation through expert consultation. Abbreviation: NGS, next-generation sequencing.

Each patient sample that is sequenced generates 2 parallel reports. The first represents a summary document with a limited, basic representation of the high-confidence resistance-associated mutations identified (Figure 1). The second, longer report includes the same high-confidence mutations but adds nuanced data, including lower-confidence mutations and technical details so that more experienced users can troubleshoot complex cases (Figure 2 and Supplementary Materials). The relationship between reports is demonstrated in Figure 2.

In both reports, resistance interpretations are based on likelihood ratios using data from a large globally representative database, in this case the ReSeqTB database [34]. Each resistance interpretation incorporates a confidence statement as described in the Disclaimer section of the comprehensive report (Supplementary Materials). Mutations that reach minimum confidence thresholds are included in both reports, which are adaptable to meet programmatic needs and could be amended to include an expanded set of loci, drugs, or different methodologies for defining interpretative criteria. The expectation is that as datasets grow and confidence statements become available for loci that impact new and repurposed drugs, such mutations, initially reported only in the comprehensive report due to limited confidence, would eventually be presented in the simple, 1-page report, provided in parallel, along with all other high-confidence resistance predications.

The 1-page report (Figure 1) summarizes mutations with a high likelihood of association with first- and second-line drug resistance, referring readers for expert consultation for pyrazinamide and moxifloxacin in the case of mutations with less clear treatment interpretations (Figure 2). Both reports include a Sample Details section with the minimal set of data elements, including relevant patient demographics, the laboratory that performed the test, requestor, specimen type, source, and collection date, the report date, a unique sample ID and a barcode associated with the submission, followed by an Assay Details section with information regarding methodology. Assay Details includes the sequencing methodology (eg, WGS or targeted sequencing), instrumentation used, analytic pipeline and version, and the reference genome. The Final Result section consists of an easy-to-locate, plain-language summary regarding *Mtb* identification and predicted drug resistance. In the example provided, the sample was positive for *Mtb* with mutations predicted to cause phenotypic resistance to isoniazid, rifampin, capreomycin, kanamycin, ofloxacin/levofloxacin, and moxifloxacin. Additional information regarding lineage and drug susceptibility results are provided in the latter sections of the report.

Preference was given to include the complete drug name instead of abbreviations. Each drug for which a gene target was evaluated is included with a categorical interpretation (resistant, susceptible, low-level resistance or intermediate, expert consultation advised, unclassified, or failure of the test to evaluate a particular drug due to low coverage at that site). Where the test failed or only specific loci were evaluated, the categorical results for interpretation might be “assay failed,” “unsuccessfully evaluated,” or “no test results.” All gene targets evaluated should be included and provide the specific nucleotide change and position(s) or amino acid change(s) using the 3-letter abbreviation with its associated codon, as applicable, along with the frequency of the reported mutation among the reads examined (ie, allele %). The *Mtb* genomic position numbering system should be used (rather than *E. coli* numbering) to avoid confusion. Workshop participants did not define a specific threshold for a resistant allele to be reported since this will differ depending on the gene of interest, the sequencing chemistry used, and the sequencing technology used. Indels are indicated in the report with the specific insertion or deletion provided.

The Comments column of the Drug Susceptibility section provides additional details including an indication that “expert consultation advised,” when molecular results indicate a more complicated interpretation (eg, heteroresistance, conflicting in vitro data, low-level resistance conferring mutations, or failure to sequence a specific gene target). Workshop participants indicated that the simplified report should focus on providing information for high-confidence mutations only, with recommendation for consultation whenever results with a more ambiguous interpretation are encountered.

The comprehensive report (Supplementary Materials) contains all information from the 1-page report but provides additional details including mutations with less definitive interpretations. This report would be relevant to expert laboratorians, researchers, and healthcare providers who could interpret complex sequencing data in order to clarify ambiguous or uncommon results when expert consultation was advised, including lower confidence mutations, mutations in loci of interest with insufficient data to generate a resistance prediction, and depth and coverage statistics to contextualize findings (Figure 2). In contrast to the 1-page report in which only high-confidence mutations are reported, all identified mutations are presented as confidence graded, showing association with resistance provided by the likelihood ratio. In the example provided (Figure 2), the *pncA* mutation T416C (amino acid change Val139Ala) is listed in the expanded report with a comment that although the mutation is known to disrupt pyrazinimidase activity, insufficient data are available to determine clinical impact on pyrazinamide use. In addition, some rare mutations may be more prevalent in certain geographical settings where local expertise may be used to contribute to the body of knowledge. This level of detail was not provided in the 1-page report and would allow the expert adjudicator to confirm pyrazinamide drug resistance. Additionally, the expanded report would specify resistance predictions with reference to different test concentrations. In Figure 2, the *gyrA* C269T (Ala90Val) mutation is reported as a high-confidence mutation. However, an interpretation of intermediate resistance and a comment indicating that at least low-level phenotypic resistance is predicted (≥0.5 μg/mL in liquid culture) suggest the potential to use a higher fluoroquinolone dose to treat the patient [35]. Workshop participants felt that making this level of information available, when possible, for laboratory reporting would be critical for aiding clinical decision making.

The comprehensive report also includes 2 additional sections. The section on “Resistance Predictions for Other TB Drugs” provides data for new or repurposed antituberculosis drugs (eg, bedaquiline, clofazimine, delamanid, and linezolid). This section is intended for informational purposes based on research and periodic review of published literature due to the lack of clinical corroborations and insufficient culture-based phenotypic data for these drugs. Nevertheless, the widespread use of WGS for *Mtb* at the global level is anticipated to provide sufficient data in the near future in order to improve the understanding of the determinants of resistance for these drugs. Optimally, updates to this section after review of new advancements would occur through global consensus. However, details regarding this process were beyond the scope of workshop objectives. Technical information in the Depth and Coverage Details section includes information relevant for understanding mutation frequency that could aid interpretation during an expert consultation. The final Disclaimer section might include relevant performance information about the methodology used and information about statistical tests used to determine confidence of the association of mutations to resistance.

## NEXT STEPS

Before broadly implementing these standards, the proposed reporting format needs to be translated into multiple languages, beta tested, and piloted in different use case settings. Gene targets included in the report could potentially change and might be determined by scheduled, iterative reviews of databases such as the ReSeqTB Data Platform [21, 34]. To guide changes, consideration of loci, interpretative criteria, and confidence levels for each mutation’s association with resistance, derived from a systematic literature review of correlations with phenotypic susceptibility test results, homoplasy, MIC values, functional genetic studies, and clinical outcomes, would be appropriate [20]. Governance for any structured reviews needs to be established. Although a good faith effort has been made to collect broad input, additional perspectives from field implementation are crucial to ensure usability and frame the terminology in the Comments section. Endorsement by entities including WHO would help ensure the sustainability of the proposed formats and help standardize NGS result reporting as access expands in global markets. In addition, this proposed model would need to be aligned with present and future commercial NGS-based diagnostics. Any reporting framework must be accompanied by education and training curricula to ensure that laboratorians, clinicians, healthcare workers, epidemiologists, and other stakeholders develop a clear understanding of the limitations of testing methodologies, the types of information provided, the format, and the interpretive comments. For clinicians, case-based learning is critical and should be customized for a range of expertise. Aligning reporting with treatment guidelines and clear avenues for expert consultation will be crucial. Consideration must also be given for combined use of phenotypic and NGS-based DST in settings where both are available. Ideally, this reporting framework could be incorporated into electronic systems linked with bioinformatic pipelines. Additional modifications may be required for this reporting format, but we believe this proposed reporting framework for clinical use of NGS data represents a step forward with real potential for global acceptance and could be used as a template to address antimicrobial resistance for other pathogens.

## Supplementary Data

Supplementary materials are available at *Clinical Infectious Diseases* online. Consisting of data provided by the author to benefit the reader, the posted materials are not copyedited and are the sole responsibility of the author, so questions or comments should be addressed to the corresponding author.

ciz219_suppl_Supplementary_Figure

ciz219_suppl_Supplementary_Material

## Notes

***Acknowledgments.*** The February 2016 workshop was jointly sponsored and convened by the Critical Path Institute and the Division of AIDS, the National Institute of Allergy and Infectious Diseases (NIAID), and the National Institutes of Health (NIH). The September 2017 workshop was jointly sponsored and convened by the Critical Path Institute and FIND diagnostics. Workgroup members who assisted in developing the agenda for the workshops include David Alland, Lori Armstrong, Alicia Chou, Katja Einer, Astrid Ferlinz, Maria Giovanni, Nazir Ismail, Karen Lacourciere, Arne Materna, Courtney Maus, Jamie Posey, Anne Purfield, Uma Devi Ramalingam, Anita Suresh, and Naomi Thomson. The authors thank all the participants, whose attendance helped shape the templates.

***Disclaimer.*** Use of trade names is for identification only and does not constitute endorsement by the US Department of Health and Human Services, the US Public Health Service, the US Centers for Disease Control and Prevention, or the NIH. The findings and conclusions in this report are those of the authors and do not necessarily represent the position of the US government or any other funding agency. The authors alone are responsible for the views expressed in this publication, and they do not necessarily represent the decisions or policies of the World Health Organization (WHO).

***Financial support.*** J. A. T. was supported by the NIAID of the NIH (grant K23AI135102); the NIH Office of the Director; Fogarty International Center; Office of AIDS Research; National Cancer Center; National Heart, Blood, and Lung Institute; and the NIH Office of Research for Women’s Health through the Fogarty Global Health Fellows Program Consortium comprised of the University of North Carolina, Johns Hopkins, Morehouse, and Tulane (R25TW009340), the Johns Hopkins University School of Medicine Clinician Scientist Career Development Award (NIH NIAID, R21AI122922), FIND, and the Gilead Foundation. FIND received support from the Bill & Melinda Gates Foundation for this study (OPP1115209). T. C. R. was supported by the NIAID of the NIH (grant R21AI135756) and received salary support from FIND. J. L. G. is supported by the Canada Research Chairs program and the Michael Smith Foundation for Health Research Scholar Awards program. T. M. W. is a National Institute for Health Research Academic Clinical Lecturer. M. Z. is a staff member of the WHO. M. S. is supported by a grant from the Bill & Melinda Gates Foundation (OPP1115887) for this study.

***Potential conflicts of interest.*** Dr. Rodwell reports funding for salary support from the Foundation for Innovative New Diagnostics, during the conduct of the study. In addition, Dr. Rodwell has a patent (SD2015-079) issued. All authors have submitted the ICMJE Form for Disclosure of Potential Conflicts of Interest. Conflicts that the editors consider relevant to the content of the manuscript have been disclosed.
