# Supplementary material for: Building the Framework for Standardized Clinical Laboratory Reporting of Next-generation Sequencing Data for Resistance-associated Mutations in Mycobacterium tuberculosis Complex
Source: Clin Infect Dis. 2019 Mar 18;69(9):1631–3. doi: 10.1093/cid/ciz219 (PMC6792097; doi:10.1093/cid/ciz219)
Supplement: ciz219_suppl_Supplementary_Figure [file ciz219_suppl_supplementary_figure.pdf]

Barcode XXXXXXXXXXXXXXXXXXXXXX

MYCOBACTERIUM TUBERCULOSIS SEQUENCING REPORT

Sample Details

Patient Name	JOHN DOE	Patient ID	12345678910
Birth Date	2000-JAN-01	Location	SOMEPLACE
Sample Type	SPUTUM	Sample Collection Date	2016-DEC-25
Sample Source	PULMONARY	Sequenced From	CULTURED ISOLATE (LJ)
Sample ID	A12345678	Sample Received Date/Time	2017-JAN-02, 12:22
Laboratory Technician	TECHNICIAN NAME	Report Date/Time	2017-JAN-05, 11:45
Requested By	REQUESTER NAME	Requester Contact	REQUESTER@EMAIL.COM

Assay Details

Sequencer	ILLUMINA HISEQ 2500	Method	WHOLE GENOME SEQUENCING
Pipeline	RESEQTB V.3.2C (https://platform.reseqtb.org)	Reference	H37RV (NC_000962.3)

Final Result

The sample was positive for ***Mycobacterium tuberculosis.*** It is resistant to isoniazid, rifampin, capreomycin, and kanamycin. It has intermediate resistance to ofloxacin and moxifloxacin. Expert consultation advised for pyrazinamide.

Lineage

Mycobacterium tuberculosis, lineage 2.2.1 (**East-Asian Beijing**).

Drug Susceptibility

Resistance is reported when a resistance-conferring mutation is detected in: *embB, gidB, gyrA, gyrB, inhA, katG, mshA, pncA, rpoB, rpsL, rrs*, or *tlyA*.[1] **No mutation detected does not exclude the possibility of resistance.**

☐ No mutations detected

☐ Multi-drug resistance predicted

☑ **Extensive drug resistance predicted**

Resistance Predictions for First Line TB Drugs

Interpretation	Drug	Gene Target (Codon Change, Amino Acid Change, Allele %)	Confidence in Resistance Association	Comments
Resistant	Isoniazid	*katG* (G944C, Ser315Thr, 100%)	High	
		inhA		No mutation detected
		mshA		No mutation detected
	Rifampin	*rpoB* (C1349T, Ser450Leu, 100%)	High	Rifabutin resistance likely
Susceptible	Ethambutol	*embB*		No mutation detected
Expert Consultation Advised	Pyrazinamide	*pncA* (T416C, Val139Ala, 98%)	Minimal	Mutation known to disrupt enzymatic activity and functional genetics in vitro. Insufficient data to determine clinical impact of this mutation.

Barcode XXXXXXXXXXXXXXXXXXXXXX

MYCOBACTERIUM TUBERCULOSIS SEQUENCING REPORT

Sample Details

Patient Name	JOHN DOE	Patient ID	12345678910
Birth Date	2000-JAN-01	Location	SOMEPLACE
Sample Type	SPUTUM	Sample Collection Date	2016-DEC-25
Sample Source	PULMONARY	Sequenced From	CULTURED ISOLATE (LJ)
Sample ID	A12345678	Sample Received Date/Time	2017-JAN-02, 12:22
Laboratory Technician	TECHNICIAN NAME	Report Date/Time	2017-JAN-05, 11:45
Requested By	REQUESTER NAME	Requester Contact	REQUESTER@EMAIL.COM

Resistance Predictions for Second Line TB Drugs

Interpretation	Drug	Gene Target (Codon Change, Amino Acid Change, Allele %)	Confidence in Resistance Association	Comments
Resistant	Capreomycin	*rrs* (C1402T, NA, 97%)	High	100% of isolates with this mutation were capreomycin resistant in the ReSeqTB database.
		tlyA		No mutation detected
	Kanamycin	*rrs* (C1402T, NA, 97%)	High	
Intermediate	Ofloxacin	*gyrA* (C269T, Ala90Val, 14%)	High	Ofloxacin and levofloxacin resistance predicted. At least low-level resistance predicted (≥0.5 µg/mL in MGIT Culture)
		gyrB		No mutation detected
	Moxifloxacin	*gyrA* (C269T, Ala90Val, 14%)	High	At least low-level resistance predicted (≥0.5 µg/mL in MGIT Culture)
		gyrB		No mutation detected
Susceptible	Amikacin	*rrs*		The C1402T mutation is associated with resistance to other injectable drugs including kanamycin and capreomycin. This mutation has not been associated with resistance to amikacin in the ReSeqTB database.
	Ethionamide	*inhA*		No mutation detected
	Streptomycin	*gidB*		No mutation detected
		rpsL		No mutation detected
		rrs		The C1402T mutation is associated with resistance to other injectable drugs including kanamycin and capreomycin. This mutation has not been associated with resistance to streptomycin in the ReSeqTB database.

MYCOBACTERIUM TUBERCULOSIS SEQUENCING REPORT

Sample Details

Patient Name	JOHN DOE	Patient ID	12345678910
Birth Date	2000-JAN-01	Location	SOMEPLACE
Sample Type	SPUTUM	Sample Collection Date	2016-DEC-25
Sample Source	PULMONARY	Sequenced From	CULTURED ISOLATE (LJ)
Sample ID	A12345678	Sample Received Date/Time	2017-JAN-02, 12:22
Laboratory Technician	TECHNICIAN NAME	Report Date/Time	2017-JAN-05, 11:45
Requested By	REQUESTER NAME	Requester Contact	REQUESTER@EMAIL.COM

Resistance Predictions for Other TB Drugs

Interpretation	Drug	Gene Target (Codon Change, Amino Acid Change, Allele %)	Confidence in Resistance Association	Comments
Insufficient Data for Resistance Prediction	Bedaquiline	*atpE*		No mutation detected
		mmpR (G198Insertion, Frameshift, 98%)	Insufficient Data	Insertion of duplicate G: CGGGGGGG<br>Gene target has shown *in vitro* resistance to bedaquiline and clofazimine in the literature.
	Clofazimine	*mmpR* (G198Insertion, Frameshift, 98%)	Insufficient Data	Insertion of duplicate G: CGGGGGGG<br>Gene target has shown *in vitro* resistance to bedaquiline and clofazimine in the literature.
	Delamanid	*ddn*		No mutation detected
		fbiA		No mutation detected
		fbiB		No mutation detected
		fbiC		No mutation detected
		fgd1		No mutation detected
	Linezolid	*rplC* (T460C, Cys154Arg, 97%)	Insufficient Data	Gene target has shown *in vitro* resistance to linezolid in the literature.
		rrl		No mutation detected

Barcode XXXXXXXXXXXXXXXXXXXXXX

MYCOBACTERIUM TUBERCULOSIS SEQUENCING REPORT

Sample Details

Patient Name	JOHN DOE	Patient ID	12345678910
Birth Date	2000-JAN-01	Location	SOMEPLACE
Sample Type	SPUTUM	Sample Collection Date	2016-DEC-25
Sample Source	PULMONARY	Sequenced From	CULTURED ISOLATE (LJ)
Sample ID	A12345678	Sample Received Date/Time	2017-JAN-02, 12:22
Laboratory Technician	TECHNICIAN NAME	Report Date/Time	2017-JAN-05, 11:45
Requested By	REQUESTER NAME	Requester Contact	REQUESTER@EMAIL.COM

Depth and Coverage Details

Locus of Interest[1]	# Reads Mapped (Depth of Coverage)	Proportion Covered (Coverage Width %)	*Mutation Frequency (% Alternate Allele)*
atpE	79	100	-
ddn	97	99.9	-
embB	88	98.8	-
fbiA	87	100	-
fbiB	91	100	-
fbiC	75	100	-
fgd1	88	100	-
gyrA	73	100	Ala90Val (13.6%*)
gyrB	76	100	-
inhA	91	100	-
katG	90	99.9	Ser315Thr (100%)
mmpR	62	100	G193Insertion (98.4%)
pncA	62	100	Val139Ala (98.4%)
rplC	78	99.9	Cys154Arg (97.4%)
rpoB	83	100	Ser450Leu (100%)
rrl	82	100	-
rrs	59	99.9	C1402T (96.6%)
tlyA	58	98.5	-

*Hetero-resistance is defined in this report as a proportion of reads for a given allele between 10% and 90%.

[1]Loci of interest derived from ReSeqTB Data Platform and from Miotto P, et al. Eur Respir J. 2017 PMID: 29284687

Disclaimer

Low frequency hetero-resistance below the limit of detection by sequencing may affect typing results. The interpretation provided is based on the current understanding of genotype-phenotype relationships. All results reference the *M. tuberculosis* mutation numbering system which differs from the *E. coli* numbering system. For rpoB add 81 to amino acid position to calculate the equivalent *E. coli* position. For gyrB subtract 7 amino acid positions to calculate the equivalent *E. coli* position.

Confidence is reported based on likelihood ratio value relating the mutation to resistance in the ReSeqTB database as follows:
- High – LR ≥ 10, high confidence that the mutation confers or is associated with resistance
- Moderate – LR ≥ 5 and <10, additional data desirable to improve evidence that the mutation confers or is associated with resistance
- Low – LR ≥ 1 and < 5, inconclusive evidence that the mutation confers or is associated with resistance. Additional data required.
- Insufficient Data – LR < 1, No evidence of association between mutation and resistance

Authorized By

Name	AUTHORIZER NAME	Position	LAB SUPERVISOR
Signature		Date	2017-JAN-05
Reporting Laboratory	LAB NAME	LAB ADDRESS	LAB PHONE NUMBER
